# New Books

**Published:** 2005-02

**A Guide to Methods in the Biomedical Sciences**

Ronald B. Corley

New York:Springer-Verlag, 2005. 166 pp. ISBN: 0-387-22844-6, $69.95

**A Land on Fire: The Environmental Consequences of the Southeast Asian Boom**

James David Fahn

New York:Basic Books, 2004. 366 pp. ISBN: 0813340535, $27.50

**An Introduction to Bioinformatics Algorithms**

Neil C. Jones, Pavel A. Pevzner

Cambridge, MA:MIT Press, 2004. 448 pp. ISBN: 0-262-10106-8, $55

**Cell Surface Receptors: A Short Course on Theory and Methods, 3rd ed.**

Lee E. Limbird

New York:Springer-Verlag, 2004. 218 pp. ISBN: 0-387-23080-7, $85

**Computational Biology of Cancer**

Dominik Wodarz, Natalia Komarova

Hackensack, NJ:World Scientific, 2005. 250 pp. ISBN: 981-256-027-0, $48

**Computational Modeling of Genetic and Biochemical Networks**

James M. Bower, Hamid Bolouri, eds.

Cambridge, MA:MIT Press, 2004. 390 pp. ISBN: 0-262-52423-6, $35

**Death Receptors in Cancer Therapy**

Wafik S. El-Deiry

Totowa, NJ:Humana Press, Inc., 2004. 400 pp. ISBN: 1-58829-172-3, $165

**Encyclopedia of Biostatistics, 2nd ed.**

Peter Armitage

Hoboken, NJ:John Wiley & Sons, Inc., 2005. 6,100 pp. ISBN: 0-470-84907-x, $2995

**Environmental Chemistry: Chemistry of Major Environmental Cycles**

Teh Fu Yen

Hackensack, NJ:World Scientific, 2005. 650 pp. ISBN: 1-86094-474-4, $76

**Environmentalism and the Technologies of Tomorrow**

Robert Olson, David Rejeski, eds.

Washington, DC:Island Press, 2004. 184 pp. ISBN: 1-55963-765-x, $45

**Gene Regulation and Metabolism: Post-Genomic Computational Approaches**

Julio Collado-Vides, Ralf Hofestädt, eds.

Cambridge, MA:MIT Press, 2004. 320 pp. ISBN: 0-262-53268-9, $28

**Hormonal Carcinogenesis IV**

Jonathan J. Li, Sara A. Li, Antonio Llombart-Bosch, eds.

New York:Springer-Verlag, 2005. 550 pp. ISBN: 0-387-23783-6, $169

**Industrial Proteomics: Applications for Biotechnology and Pharmaceuticals**

Daniel Figeys

Hoboken, NJ:John Wiley & Sons, Inc., 2005. 320 pp. ISBN: 0-471-45714-0, $79.95

**Methods of Microarray Data Analysis IV**

Jennifer S. Shoemaker, Simon M. Lin, eds.

New York:Springer-Verlag, 2005. 270 pp. ISBN: 0-387-23074-2, $109

**Microarray Technology and Its Applications**

U.R. Müller, D.V. Nicolau, eds.

New York:Springer-Verlag, 2005. 379 pp. ISBN: 3-540-22931-0, $129

**Nanotechnology: Environmental Implications and Solutions**

Louis Theodore, Robert G. Kunz

Hoboken, NJ:John Wiley & Sons, Inc., 2005. 392 pp. ISBN: 0-471-69976-4, $99.95

**Probabilistic Modeling in Bioinformatics and Medical Informatics Series: Advanced Information and Knowledge Processing**

Dirk Husmeier, Richard Dybowski, Stephen Roberts, eds.

New York:Springer-Verlag, 2005. 505 pp. ISBN: 1-85233-778-8, $89.95

**Proteomics Today: Protein Assessment and Biomarkers Using Mass Spectrometry, 2D Electrophoresis, and Microarray Technology**

Mahmoud H. Hamdan, Pier G. Righetti, Dominic M. Desiderio, Nico M. Nibbering

Hoboken, NJ:John Wiley & Sons, Inc., 2005. 448 pp. ISBN: 0-471-64817-5, $89.95

**The Bhopal Saga: Causes and Consequences of the World’s Largest Industrial Disaster**

Ingrid Eckerman

Andhra Pradesh, India:Universities Press, 2004. 304 pp. ISBN: 81-7371-515-7, $25

**The Carcinogenic Effects of Polycyclic Aromatic Hydrocarbons**

Andreas Luch, ed.

Hackensack, NJ:World Scientific, 2005. 740 pp. ISBN: 1-86094-417-5, $178

**The Portland Edge: Challenges and Successes in Growing Communities**

Connie P. Ozawa, ed.

Washington, DC:Island Press, 2004. 321 pp. ISBN: 1-55963-687-4, $60

